# Validation of Siemens T2* inline WIP package for quantification of cardiac and hepatic iron loading at 1.5T and 3T

**DOI:** 10.1186/1532-429X-16-S1-P323

**Published:** 2014-01-16

**Authors:** Mohammed H Alam, Arun J Baksi, Taigang He, Gillian C Smith, Cemil Izgi, Ricardo Wage, Peter Drivas, Andreas Greiser, Bruce S Spottiswoode, David Firmin, Dudley J Pennell

**Affiliations:** 1 NIHR Cardiovascular Biomedical Research Unit, Royal Brompton Hospital, London, UK; 2 Imperial College London, London, UK; 3 Siemens AG Healthcare Sector, Erlangen, Germany; 4 Siemens Healthcare USA Inc., Malvern, Pennsylvania, USA

## Background

The ability of T2* cardiovascular magnetic resonance (CMR) to identify cardiac iron loading has facilitated a dramatic reduction in mortality in patients with iron overload. There remains a worldwide need for improved access to iron evaluation. One route to achieving this would be simple in-line T2* analysis. We compared our validated T2* methods which use Royal Brompton Hospital (RBH) T2* sequences with analysis by CMRtools against a novel work-in-progress (WIP) sequence and inline T2* analysis.

## Methods

22 healthy volunteers and 78 patients were recruited (thalassaemia major 39, sickle cell disease 15, hereditary hemochromatosis 10, other iron overload conditions 14) who were referred for routine iron assessment (53 male, aged 13 to 81 years). A 1.5T study (MAGNETOM Avanto, Siemens AG Healthcare Sector, Erlangen, Germany) was performed on all subjects, from whom a subset of 50 underwent an additional 3T study (MAGNETOM Skyra). The same mid-ventricular short axis cardiac slice and transaxial slice through the liver were used to acquire both RBH T2* images and WIP T2* maps for each scan. Cardiac white blood (WB) and black blood (BB) sequences were acquired. All data acquisition and ROI based analysis was performed by a single observer.

## Results

**Figure 1:**
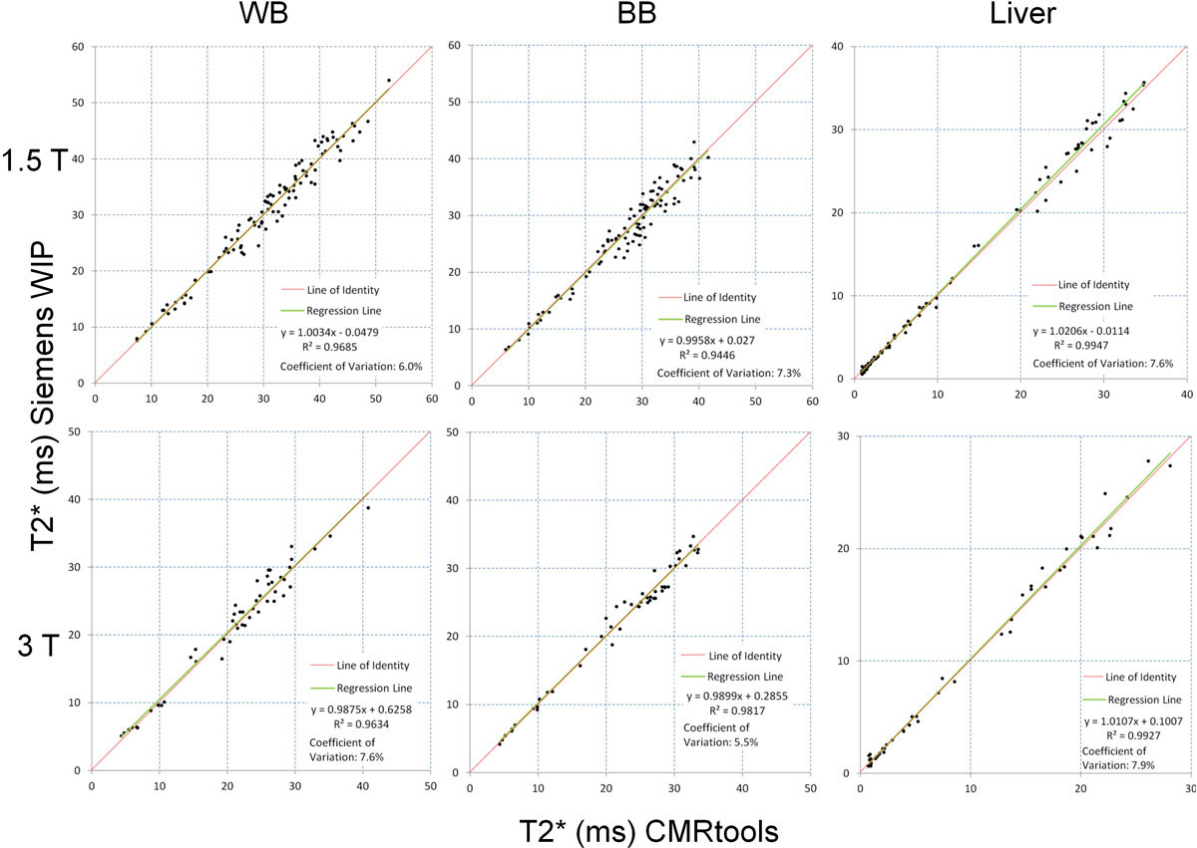
Plots showing correlation of RBH T2* with Siemens WIP T2* for white blood (WB), black blood (BB) and liver at 1.5T and 3T respectively.

At 1.5T, liver T2* values ranged from 0.8-33 ms (median 5.1 ms) and cardiac T2* values from 6.6-49 ms (median 31 ms). There was good agreement between RBH-CMRtools T2* and Siemens T2* WIP maps with results close to the line of identity and linear regression close to 1 (Figure 1); R^2 ^values for WB, BB and liver T2* were 0.969, 0.945 and 0.995 respectively at 1.5T and 0.963, 0.982 and 0.993 respectively at 3T. The coefficient of variation was 5.5-7.6% across techniques at 1.5 T and 5.5-7.9% at 3T. Accurate delineation of the septum was difficult on some T2* WIP maps due to artefacts. The inability to manually correct for noise by truncation of erroneous later echo times lead to some overestimation of T2* using the WIP technique compared to RBH-CMRtools.

## Conclusions

The Siemens WIP T2* mapping sequence and analysis performed well against the standard RBH-CMRtools T2* package at both 1.5T and 3T. Inline T2* mapping in combination with a simple ROI analysis has the potential to improve global access to iron assessment.

## Funding

This research was supported by the NIHR Cardiovascular Biomedical Research Unit at Royal Brompton & Harefield NHS Foundation Trust and Imperial College London.

